# Development of a high-yield Rabbit line for enhanced animal pharming

**DOI:** 10.1186/s40659-025-00653-y

**Published:** 2025-12-10

**Authors:** Jun Song, Dongshan Yang, Lingjie Kong, Li-Kuang Tsai, Jifeng Zhang, Y. Eugene Chen, Ruby Yanru Tsai, Jie Xu

**Affiliations:** 1 Center for Advanced Models for Translational Sciences and Therapeutics, University of Michigan Medical Center, Ann Arbor, MI 48109 USA; 2 Applied StemCell, Inc.,, 521 Cottonwood Dr. Suite #111, Milpitas, CA 95035 USA

**Keywords:** Animal pharming, Rabbits, CRISPR/Cas9, CSN2 gene promoter

## Abstract

Animal pharming involves producing recombinant protein drugs using transgenic animals. The United States Food and Drug Administration (FDA) has approved certain drugs produced in the milk of transgenic Rabbits. Traditionally, these pharming Rabbits have been developed using conventional transgenic technology, which often results in an unpredictable success rate, uncontrollable transgene insertion sites, varying copy numbers, and generally low recombinant protein yields, typically 1–2 g/L or lower. We hypothesized that utilizing the promoter of a native major milk protein gene to drive transgene expression could significantly enhance yield. To test this, we developed a rabbit line that expresses tdTomato under the control of the CSN2 gene promoter, responsible for encoding β-casein, the most abundant protein in Rabbit milk. We successfully generated knock-in founder Rabbits using CRISPR/Cas9-mediated knock-in technology, augmented by the homology-directed repair (HDR)-promoting small molecule RS-1. These founder Rabbits were able to transmit the knock-in allele to their offspring, producing both heterozygous and homozygous tdTomato knock-in Rabbits. Remarkably, the recombinant protein yield reached 15–20 g/L in the milk of homozygous animals. Our work demonstrates a promising strategy to enhance recombinant protein production in Rabbit pharming.

**Supplementary Information:**

The online version contains supplementary material available at 10.1186/s40659-025-00653-y.

## Introduction

Animal pharming utilizes genetically modified animals to produce recombinant proteins in various bodily fluids such as milk and blood [1–4]. Several recombinant proteins have been successfully produced in the milk of transgenic animals [3]. Rabbits are a key species in animal pharming. Of the three U.S. Food and Drug Administration (FDA)-approved drugs derived from transgenic milk (Supplementary Fig. 1A), the first one is produced in goats, and the other two are produced in Rabbits. Of these two, recombinant C1 inhibitor, marketed as Ruconest, received approval in the European Commission (EC) in 2010 and in the United States (U.S.) in 2014, and recombinant activated human coagulation Factor VII, known as Sevenfact, was approved in the U.S. in 2020.

Most pharming Rabbit lines, including those used to produce Ruconest and Sevenfact, are created using the conventional transgenic technology of pronuclear (PN) DNA microinjection. This method, developed nearly half a century ago [5], offers limited control over the transgene's integration site and copy number, leading to unpredictable expression levels often below the industrial threshold of 2 g/L. A comprehensive review by Wang et al. in 2013 [3] indicated that numerous therapeutic proteins have been expressed in Rabbit milk using different promoters—primarily β-casein, WAP, and αS1 from various species—via PN microinjection, with varying degrees of success in terms of yield. Of the 23 studies that reported recombinant protein yields, 61% achieved yields below 1.0 g/L, and over one-third reported yields are below 0.1 g/L (Supplementary Fig. 1B). Only 17% reached yields of greater than 2.0 g/L, and a mere 4% exceeded 5 g/L. This trend has persisted; for example, in 2019, He et al. reported a yield of 0.95 g/L for recombinant human plasminogen activator (rhPA) in Rabbit milk [6]. Similarly, a 2022 study by the same group found the yield for recombinant desmodus rotundus salivary plasminogen activator alpha1 (rDSPAα1) to be 1.2 g/L in the milk [7]. In both instances, the Rabbits were produced using conventional transgenic methods.

We hypothesized that the precise gene knock-in capabilities of modern gene editing tools such as clustered regularly interspaced short palindromic repeats and CRISPR-associated protein 9 (CRISPR/Cas9) could significantly enhance Rabbit pharming in three key aspects: (i) allowing precise control over the insertion site of the transgene; (ii) enabling control of the transgene copy number; and (iii) through the above two, improving recombinant protein yield, for example, by placing transgene expression under the control of the promoter of an endogenous major milk protein gene.

In this study, by using CRISPR/Cas9 mediated knock-in, we developed a Rabbit line that expresses high levels of the recombinant protein tdTomato driven by the endogenous promoter of the CSN2 gene, which encodes a major milk protein β-casein [8]. Our work illustrates a viable strategy for enhancing recombinant protein yield in Rabbit pharming.

## Materials and methods

### Animals

New Zealand White (NZW) Rabbits were used in the present work. The animal maintenance, care and use procedures were reviewed and approved by the Institutional Animal Care and Use Committee (IACUC) of the University of Michigan. All procedures were carried out in accordance with the approved guidelines.

## Cas9 protein, single-guide RNA (sgRNA), and knock-in donor template

The single-guide RNAs (sgRNAs), Cas9 protein and single-stranded oligodeoxynucleotides (ssODNs) were purchased from Integrated DNA Technologies (Newark, NJ, USA).

The sgRNA target sequences are shown below:

- sgRNA#1: 5′- GGTGGCTCTCGCTCTTGCAA (GGG) -3′
- sgRNA#2: 5′- GGTCCTCATTCTCGCCTGCC (TGG) -3′

The donor plasmid contains the coding sequence for a reporter transgene tdTomato, flanked by 5′ and 3′ homologous arms (HAs), as well as a signal peptide sequence to make the recombinant protein secreted. In addition, with a long-term goal to streamline the pharming Rabbit production in mind, we included a PhC31 attP site at the 5′ and a Bxb attP site at the 3′ adjacent to the transgene. These attP sites are expected to facilitate uni-directional integrase (i.e., PhC31 and Bxb) mediated knock-in of new transgene to this locus in a cassette-exchange manner, a method that we have previously demonstrated in mice [9]. Supplementary Fig. 2 shows the donor plasmid layout.

## Superovulation, microinjection and embryo transfer

Rabbit embryos were collected for microinjection, as previous described [10]. Briefly, sexually matured NZW female Rabbits in the age range of 6 to 12 months-old were hormonally stimulated with subcutaneous injections of follicle-stimulating hormone (FSH, Folltropin-V, Bioniche Life Sciences, Canada) every 12 h for six times, followed by a single intravenous injection of 200 IU human chorionic gonadotropin (hCG, Chorulon, Intervet, Millsboro, DE, USA) at 12 h after the last FSH injection to induce ovulation. Immediately after the hCG injection, the female Rabbit was mated with a male Rabbit. At the same time, the recipent female animal was injected a single dosage of 200 IU hCG to induce synchronization. Eighteen to twenty hours post mating, pronuclear stage embryos were harvested and placed in manipulation medium consisting of medium 199 (M199) supplemented with 25 mM HEPES (12,350,039, Thermo Fisher Scientific, Waltham, MA, USA) and 10% Fetal Bovine Serum (FBS, 10437–028, Thermo Fisher Scientific) for microinjection.

Pronuclear microinjection was carried out similarly as previously described [10]. Briefly, 1–5 ng/μL donor DNA was injected into the male pronucleus, and a mixture containing 100 ng/μL Cas9 mRNA and 6 ng/μL sgRNA were administrated into cytoplasm. Post-injection embryos were rinsed thrice in pre-equilibrated embryo culture medium, which consisted of Earle's Balanced Salt Solution (E2888, Sigma-Aldrich, St Louis, MO, USA) supplemented with non-essential amino acids (M7145, Sigma-Aldrich), essential amino acids (B-6766, Sigma-Aldrich), 1 mM L-glutamine (25,030–081, Thermo Fisher), 0.4 mM sodium pyruvate (11,360–070, Thermo Fisher) and 10% FBS. Embryos were cultured overnight with or without 7.5 μM RS-1 (553,510, Sigma-Aldrich) treatment prior to transfer. Embryo transfer was carried out similarly as previously described [11]. Twenty to thirty embryos were surgically transferred to synchronized female through the infundibulum into both sides of the oviducts. For in vitro validation, embryos were washed and cultured in medium with/without RS-1 until blastocyst stage.

## Confirmation of gene targeting events in embryos and in animals

For in vitro validation*,* individual blastocysts were lysed and genomic DNA extracted. To get better PCR reaction, the whole genome was amplified using a REPLI-g Mini Kit (150,023, Qiagen, Germantown, MD, USA) following the manufacturer’s protocol with slight modification. Briefly, for harvesting denatured DNA, 3.5 μL Buffer D2 was added to each embryo, mixed by vortexing and centrifuged briefly. The samples were incubated on ice for 10 min. After that 3.5 μL Stop Solution was added, mixed by vortexing and centrifuged briefly. For replication, 2 μL of the denatured DNAs were added to 8 μL master mix and incubate at 30 °C for 10–16 h. Then REPLI-g Mini DNA Polymerase was inactivated by heating at 65 °C for 3 min.

To detect knock-in events in putative founder animals, ear skin tissue pieces were biopsied, genomic DNA extracted, and used for PCR using primer pairs LF1/LR1 and RF1/RR1. The LF1/LR1 primer pair amplify the left junction of the knock-in fragment, whereas the RF1/RR1 primer pair amplify the right junction of the knock-in fragment (Fig. 2A). The PCR products were used in agarose gel electrophoresis, then gel purified for Sanger sequencing at Eurofins Genomics (Lancaster, PA, USA) to confirm the knock-in sequences. A knock-in event will show a 1.9 kb band from the products by the LF1/LR1 primer pair, and a 1.3 kb band from products by the RF1/RR1 primer pair. Sanger sequencing traces were analyzed by Mutation Surveyor (SoftGenetics, State College, PA). The sequences of LF1/LR1 and RF1/RR1 are shown below:

- LF1: 5′- CATGGTCTTTGTGAGCCCCTGTG -3′
- LR1: 5′- ACCCTGGACTACTGCGCCCTGTTT -3′
- RF1: 5′- CCACACCTCCCCCTGAACCTGAAACAT -3′
- RR1: 5′- TCCCTTGCAAGAGCGAGAGC -3′

To genotype the F1 and later generation animals, ear skin tissue pieces were biopsied, genomic DNA extracted, and used for PCR using another pair primers L1/R1. L1/R1 spans across the knock-in fragment (Fig. 2A). The knock-in allele will show a 2995 bp band, whereas the wild-type allele will show a 981 bp band. The sequences of L1/R1 are shown below:

- L1: 5′- TGGGCCCAGGAGATGACAGTTTT -3′
- R1: 5′- GCCGGGTAGAGCTTTGGGTTTTT -3′

## Determination of the concentrations of tdTomato in Rabbit milk

Recombinant tdTomato (TP790045, Origene, Rockville, MD, USA) was utilized to create a standard curve. Briefly, the recombinant tdTomato was serially diluted to concentrations of 0 (no tdTomato), 0.135, 0.108, 0.054, 0.027, and 0.0135 g/L using milk obtained from a wild-type female Rabbit, forming the Standards. Testing samples, i.e., milk collected from pharming Rabbits, were serially diluted to 100x, 200x, and 500 × with the same wild-type Rabbit milk used for the Standards. These Standards and the testing samples were then placed in a 96-well microplate and analyzed using a GloMax Discover Microplate Reader (Promega, Madison, WI, USA). A preloaded fluorescence protocol was executed with an excitation wavelength of 520 nm and an emission range of 580–640 nm, and the Optical Density (OD) values of the Standards were measured. The OD values, along with their corresponding tdTomato concentrations of the Standards, were used to establish the standard curve. The OD values of the testing samples were used to calculate the tdTomato concentrations using the correlation formula derived from the standard curve.

## Statistical analysis

Chi-square test in Prism 9.0 (Graphpad Software, Inc., La Jolla, CA, USA) was used to calculate P values when comparing the knock-in rates between the RS-1 treatment and the control groups. Significant differences were defined as *P* < 0.05 (*). The linear correlation was analyzed by Microsoft Excel (Version 16.98, Microsoft, Redmond, WA, USA).

## Results

### Design and validation of sgRNAs to target the CSN2 locus

**Fig. 1:**
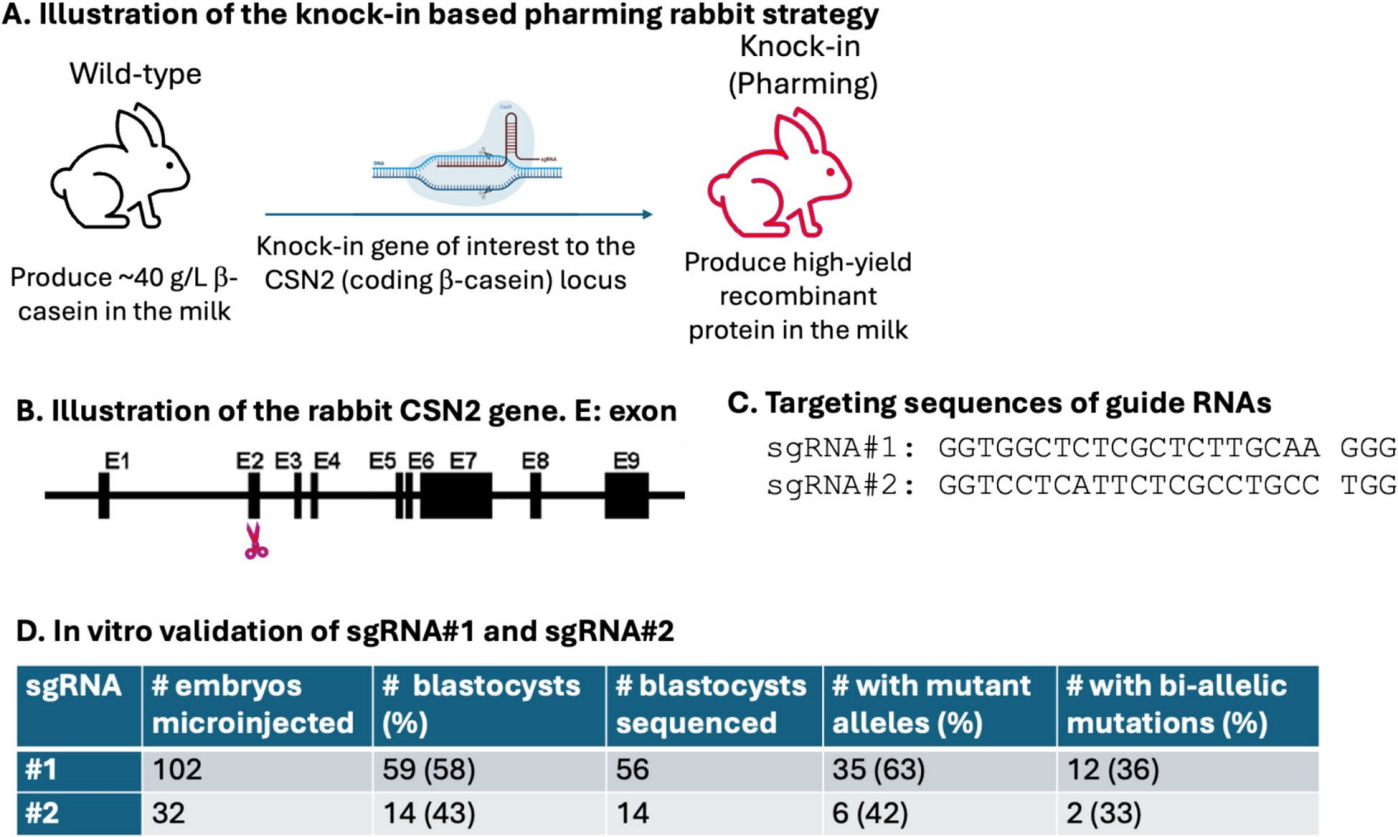
Validate the targeting guide RNAs for Rabbit CSN2 gene. **A** Illustration of the knock-in based pharming Rabbit stratety to modernize Rabbit pharming. **B** Illustration of the Rabbit CSN2 gene. E: exon. Scissors indicate the target site. **C** Guide RNA targeting sequences at E2 of the Rabbit CSN2 gene. **D** In vitro validation results of the guide RNAs targeting Rabbit CSN2

We hypothesized that a high yield of the recombinant protein can be achieved when the transgene expression is driven by the endogenous CSN2 promoter (Fig. 1A). The Rabbit CSN2 gene is located on Chromosome 15, consisting of 9 exons. It encodes β-Casein, the most abundant milk protein in Rabbits. The gene organization is illustrated in Fig. 1B. The first coding Exon is Exon 2 (E2).

First, we worked to identify a guide RNA that can efficiently target the CSN2 E2 locus. We designed two single-guide RNAs (sgRNA#1 and sgRNA#2) targeting E2 (Fig. 1C). Pronuclear stage embryos collected from 4 embryo donors were pooled and were individually injected with Cas9 mRNA and one of the sgRNAs, and cultured in vitro for 3 to 4 days until they reach the blastocyst stage. Individual blastocyst stage embryos were subjected to PCR amplification followed by Sanger sequencing to evaluate the insertion and deletion (indel) frequencies at the target site.

In total, we microinjected 102 embryos with sgRNA#1 and 32 embryos with sgRNA#2. For sgRNA#1, 63% (35/56) blastocysts contained indels at the target site (Fig. 1D). Slightly lower indel rate was achieved by sgRNA#2 (6/14, 42%). The rate of bi-allelic mutation, defined as indel formation on both alleles, is also slightly higher in embryos injected with sgRNA#1 than in those injected with sgRNA#2 (36% vs. 33%, Fig. 1D). Based on these results, sgRNA#1 was selected for subsequent knock-in work.

## Design and validation of the donor DNA

We then tested the knock-in efficiency by using the knock-in donor template and the validated sgRNA#1 in in vitro cultured embryos. A total of 195 pronuclear stage embryos collected from 10 embryo donor animals were pooled and individually microinjected with Cas9 mRNA, sgRNA#1 and the donor plasmid.

**Fig. 2:**
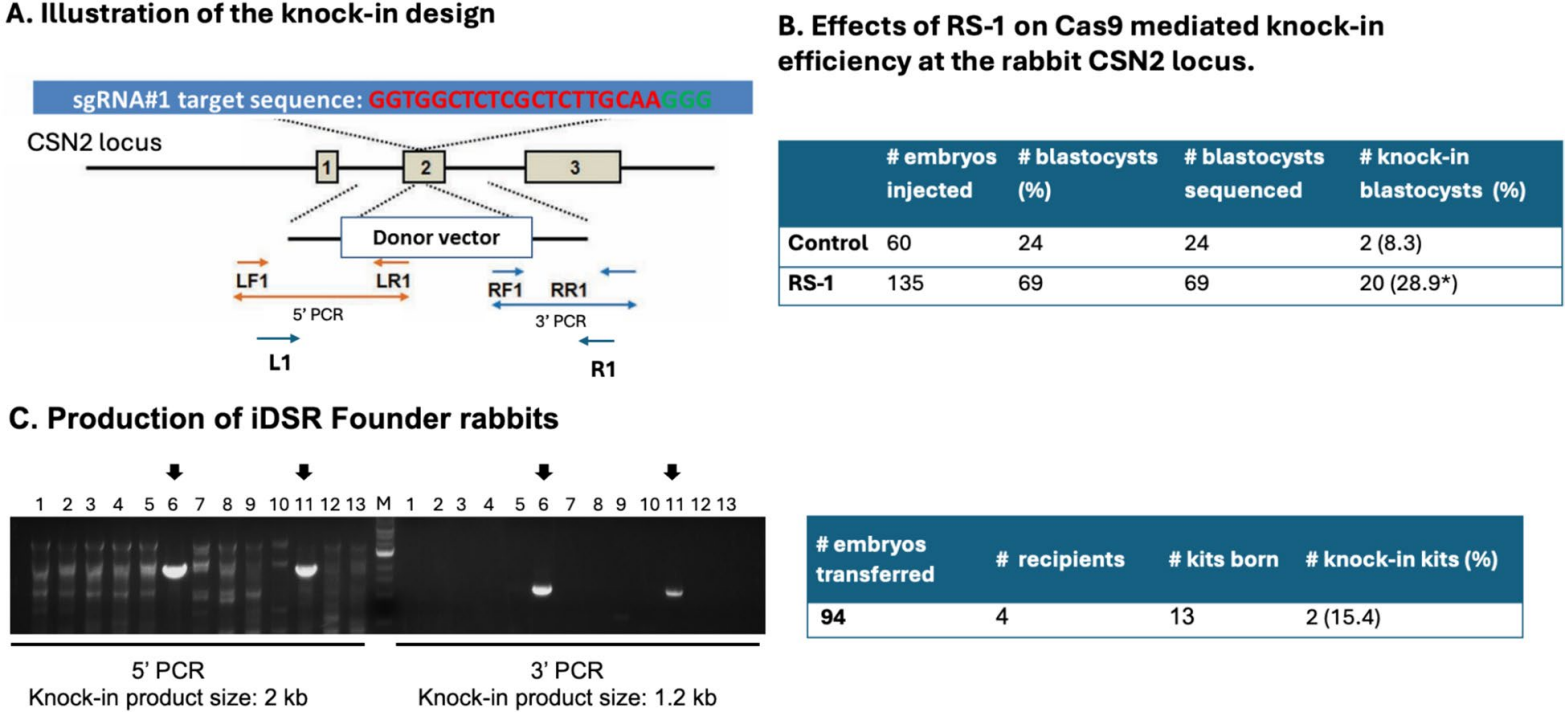
Production of the founder pharming Rabbits. **A** Illustration of the knock-in design to produce founder pharming Rabbits. LF1/LR1, RF1/RR1 and L1/R1: primer pairs used to detect knock-in events. **B** Effects of RS-1 on Cas9 mediated knock-in rates at the CSN2 locus in Rabbit embryos. **C** Left: a representative genotyping gel to identify putative pharming founder Rabbits. PCR were conducted at both 5’ junction (5’ PCR) and 3’ junction (3’ PCR). Arrows indicate putative founder Rabbits based on the band sizes. Right: summary of the knock-in founder production efficiency

In a previous work, we reported that supplementation of a homology-directed-repair (HDR)-promoting compound RS-1 in the embryo culture medium enhances CRISPR/Cas9-mediated knock-in efficiency [12]. So, we included RS-1 treatment in the experiment. After microinjection of Cas9 mRNA, sgRNA#1 and the donor template DNA to the pronuclear stage embryos, these embryos were cultured in vitro with (n = 135) or without (n = 60) the RS-1 supplementation, until reaching blastocyst stage, after which the genomic DNA were extracted and subjected to PCR amplification and gel electrophoresis to detect if any knock-in specific bands are present in each individual embryo. The left arm knock-in sequence was amplified by primer set LF1/LR1 and the right arm knock-in sequence was amplified by primer set RF1/RR1 (Fig. 2A). Embryos containing both left arm and right arm knock-in bands are considered putative knock-in.

In the group of embryos that were not treated with RS-1, 8.3% knock-in rates were achieved; whereas in the group of embryos treated with RS-1, the knock-in rates were > threefold higher at 28.9% (Fig. 2B), confirming our previous finding that RS-1 improves CRISPR/Cas9 mediated knock-in rates in Rabbit embryos [12].

## Production of founder pharming Rabbits

We proceeded to produce founder Rabbits using the validated sgRNA#1 and the donor template. Pronuclear stage embryos were collected from 4 embryo donor animals, microinjected with Cas9 mRNA, sgRNA#1 and the donor template, and transferred into the oviducts of synchronized recipient animals. In total we transferred 94 embryos to 4 recipients, 3 became pregnant and gave birth to 13 kits. Genotyping of the newborn kits was performed by PCR amplification of genomic DNA extracted from ear skin biopsies, using the corresponding primers described earlier. In addition to gel electrophoresis (Fig. 2C), we subjected the PCR products to Sanger sequencing to ensure complete sequence match with the knock-in design (Supplementary Fig. 3). The results show that 2 out of 13 (15.4%) kits are knock-in founders.

## Production of heterozygous and homozygous pharming Rabbits

**Fig. 3:**
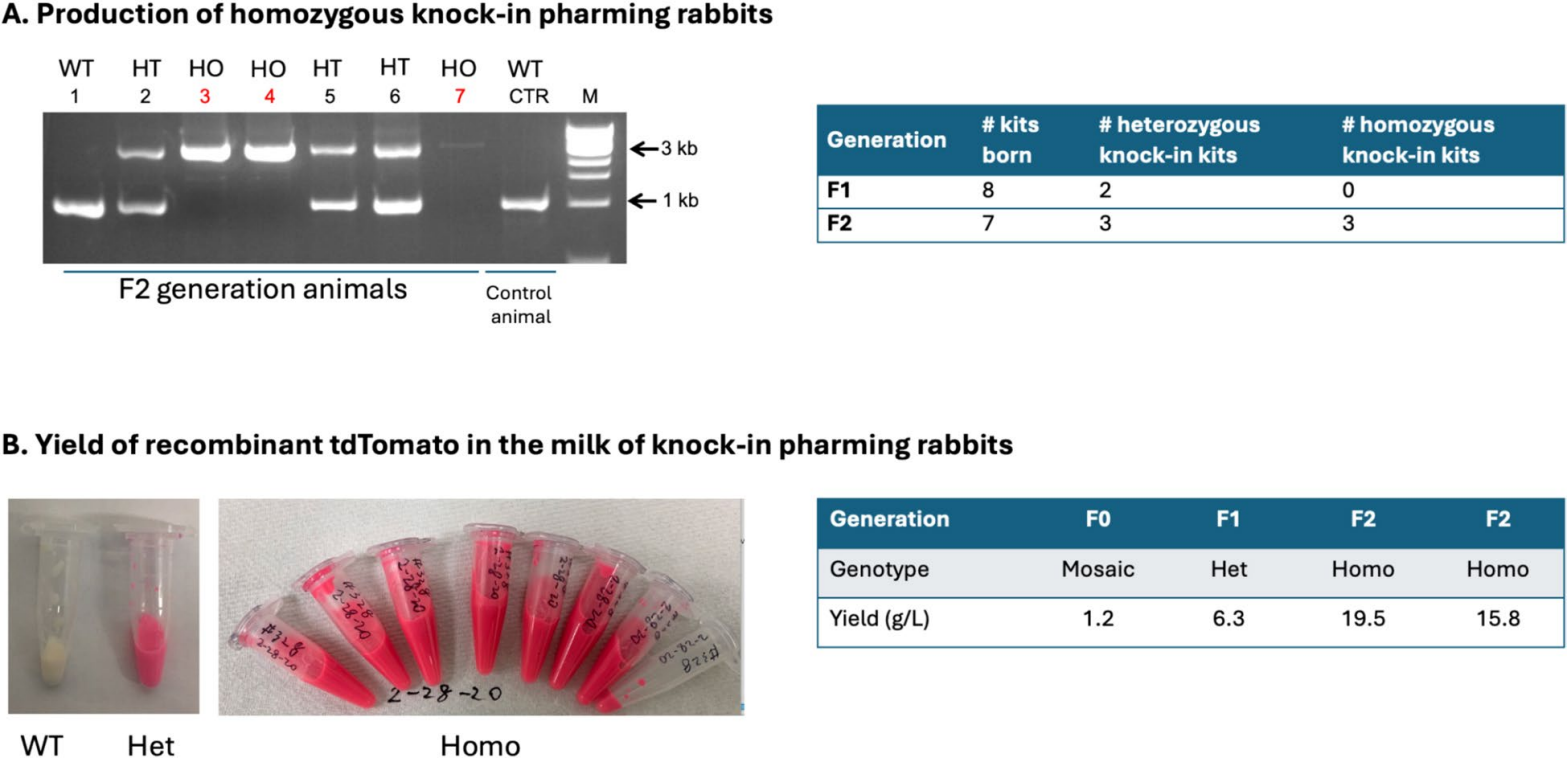
High yield of recombinant protein in the milk of pharming Rabbits.** A** Production of homozygous knock-in pharming Rabbits. Left: genotyping results of heterozygous (HT) and homozygous (HO) F2 generation pharming Rabbits. WT CTR: wild-type control. M: molecular weight. Right: summary of the breeding outcome to product F1 and F2 generation animals.** B** Yields of recombinant tdTomato in the milk of pharming Rabbits. Left: example images of harvested milk from wild-type (WT), heterozygous (Het) and homozygous (Homo) pharming Rabbits. Note the bright red color in the milk of pharming Rabbits. Right: summary of yields in the milk of F0, F1 and F2 generation Rabbits

Upon sexual maturation, the two F0 generation (i.e., founder) pharming Rabbits were each bred with a wild-type (WT) counterpart to test germline transmission of the knock-in allele. A total of eight kits were produced, among which two were confirmed to be heterozygous knock-in (F1 generation). This proved the germline transmission capacity of the pharming Rabbit founders (Fig. 3A, right panel).

After this pair of F1 generation heterozygous animals, one male and one female, reached sexual maturation, they were bred and produced seven kits. Out of these seven, one is WT, three are heterozygous and three are homozygous knock-in, as confirmed by PCR (Fig. 3A, left and right panels).

## High yield of recombinant tdTomato in the milk of pharming Rabbit

Our hypothesis is that the yield of recombinant protein under the control of the endogenous CSN2 promoter will be high. We hence tested the concentration of tdTomato in the milk collected from one F0 generation founder female, one F1 generation heterozygous female, and two F2 generation homozygous females.

We used recombinant tdTomato as the standard. We diluted it using milk from a WT Rabbit to 0 (blank), 0.135, 0.108, 0.054, 0.027, and 0.0135 g/L, and used them to establish a standard curve for the tdTomato concentration assay (Supplementary Fig. 4A).

Milk collected from heterozygous and homozygous knock-in female Rabbits were brightly red as observed by naked eyes, in contrast with the milk from a WT female (Fig. 3B, left panels). To determine the tdTomato concentrations, we serially diluted (2x, 10x, 100x, 200x, 500x) the milk from pharming Rabbits with the WT Rabbit milk that was used in establishing the standard curve, measured the OD readings and calculated the estimated concentrations.

The tdTomato concentration is low in the milk from the founder F0 female (1.2 g/L). Remarkably, the tdTomato concentrations sharply increased in the milk from the heterozygous pharming Rabbit to 6.3 g/L (Fig. 3B, right panel & Supplementary Fig. 4B). In the two homozygous animals, the tdTomato yields were further increased to 15.8 and 19.5 g/L, respectively (Fig. 3B, right panel). These data support our hypothesis that high yield of recombinant protein in the milk can be achieved if the transgene is expressed under the control of the endogenous CSN2 promoter.

## Discussion

In the present work, we developed a high-yield Rabbit line for animal pharming. In this line, the transgene encoding tdTomato is driven by the promoter of endogenous CSN2 gene, which encodes β-casein. In Rabbits, β-casein is the most abundant milk protein with a concentration greater than 40 g/L (*8*). We had hypothesized that knocking-in the transgene of interest to the CSN2 locus would elevate the yield of recombinant protein to a level comparable to that of β-casein. Indeed, our data supported this hypothesis. The tdTomato concentrations in the milk from two homozygous knock-in female Rabbits have reached 15–20 g/L, one to multiple-magnitudes higher than most reported yields in the milk of pharming Rabbits produced by the conventional transgenic method (Supplementary Fig. 1B). Our work thus demonstrates the feasibility of improving the yield of recombinant protein in pharming Rabbit milk to a new level into the 10–20 g/L range.

The conventional pronuclear DNA microinjection has been the dominant method to produce pharming animals especially in the era prior to gene editing nucleases. This method however suffers from the following: (i) the transgenic success rate is generally low; (ii) the integration site and the orientation of the transgene(s) are random; (iii) the copy number of transgene is unknown and uncontrollable. In addition, this method uses exogenously constructed promoter, which does not take advantage of highly efficient endogenous locus for recombinant protein production. These together, in our opinion, have contributed to the overall situation that the yield of pharming Rabbit milk is low.

CRISPR/Cas9, since its debut in 2013 [13], has empowered researchers to produce gene targeted animal models in species where germline transmitting embryonic stem cells are not available. Our group have established an efficient platform to produce versatile gene knockout and knock-in Rabbit models [12, 14]. In the context of Rabbit pharming, there has been very limited work to produce pharming Rabbits using CRISPR/Cas9 or other gene editing nucleases. Literature search conducted in May 2025 shows that there is only one report by Li et al. in 2022, in which they integrated rotavirus VP6 gene to the Rabbit CSN2 locus by CRISPR/Cas9. They achieved high knock-in rates (20%); however, the yield of VP6 protein in the Rabbit milk is not reported [15].

As such, our work represents the first one with recombinant protein yield data in pharming Rabbits that are produced by CRISPR/Cas9. Comparing to the conventional PN microinjection method, CRISPR/Cas9 offers the following advantages: (i) the integration site and the orientation of the transgene can now be precisely controlled; (ii) the copy number of the transgene can now be precisely controlled; (iii) the success rate of knock-in can be high (> 15%), for example, at the CSN2 locus with the augmentation by HDR promoting compound RS-1 (Fig. 2). What’s more, it allows the hijacking of the endogenous promoter (e.g., CSN2) to drive the expression of the transgene, which as shown in the present work, has led to an unprecedented high yield (15 – 20 g/L) of the recombinant protein (Fig. 3B).

With a long-term goal to streamline the production of pharming Rabbits, we purposely flanked the tdTomato transgene with two integrase recognition sequences: (i) the PhC31 attP site at the 5’; and (ii) the Bxb attP site at the 3’ (Supplementary Fig. 2). Therefore, the tdTomato knock-in Rabbits are in fact docking site ready (DSR), such that when a donor template containing the PhC31 attp and the Bxb attp sequences flanking a new transgene of interest is injected to the DSR Rabbit embryos along with PhC31 and Bxb integrases, integrase mediated gene knock-in will take place to produce new pharming Rabbit lines in a cassette exchange manner (Supplementary Fig. 5). We envision that in the future new lines of pharming Rabbits can be efficiently produced through this approach.

We want to point out some limitation and future research directions of this work. First, due to the relatively long sex maturation time of Rabbits, we did not test the production of new pharming Rabbit lines using the “DSR embryo + integrase” method, which certainly warrants experimental validation. Second, tdTomata is used in the present work to demonstrate the proof of concept. Therapeutically relevant proteins of interest, for example nanobodies and anti-bacterial peptides, should be tested in this system. Third, the yield of 15–20 g/L recombinant protein is still only half that of the endogenous β-casein (> 40 g/L) in the Rabbit milk. Whether the yield can be further improved by incorporating other endogenous regulatory elements or through other methods certainly is worthy additional research.

## Conclusion

The use of CRISPR/Cas9-mediated knock-in technology, combined with the CSN2 gene promoter, significantly enhanced recombinant protein yields in transgenic Rabbits, achieving levels over 15–20 g/L. This approach offers a promising strategy to improve protein production efficiency in animal pharming.

## Supplementary Information

Additional file1 (DOCX 1378 kb)

## Data Availability

All data supporting this study are available within this publication, and materials can be accessed through a material transfer agreement between the University of Michigan, Applied Stemcell, and the requesting party.

## Abbreviations

FDA: Food and drug administration
CRISPR/Cas9: Clustered regularly interspaced short palindromic repeats and CRISPR-associated protein 9
HDR: Homology directed repair
PN: Pronuclear
rhPA: Recombinant human plasminogen activator
rDSPAα: Recombinant desmodus rotundus salivary plasminogen activator alpha1
NZW: New Zealand white
sgRNA: Single-guide RNA
ssODNs: Single-stranded oligodeoxynucleotides
HA: Homologous arms
FSH: Follicle-stimulating hormone
hCG: Human chorionic gonadotropin
M199: Medium 199
FBS: Fetal bovine serum
PCR: Polymerase chain reaction
DNA: Deoxyribonucleic acid
RNA: Ribonucleic acid
OD: Optical density
WT: Wild type
