# Supplementary material for: Development of a high-yield Rabbit line for enhanced animal pharming
Source: Biol Res. 2025 Dec 10;58:73. doi: 10.1186/s40659-025-00653-y (PMC12696889; doi:10.1186/s40659-025-00653-y)

**Supplementary Figures**

**Title:** Development of a High-Yield Rabbit Line for Enhanced Animal Pharming

**Authors:** Jun Song ^1,#^, Dongshan Yang ^1,#^, Lingjie Kong ^2^, Li-Kuang Tsai ^1^, Jifeng Zhang ^1^, Y. Eugene Chen ^1^, Ruby Yanru Tsai ^2,*^, Jie Xu ^1,*^

**Supplementary Figures**

**Supplementary Figure 1. Rabbit is a major pharming species.** (A) Summary of FDA approved pharming drugs produced in transgenic animals’ milk. (B) Summary of yields of recombinant proteins in the milk of pharming rabbits based on reference (3).

**
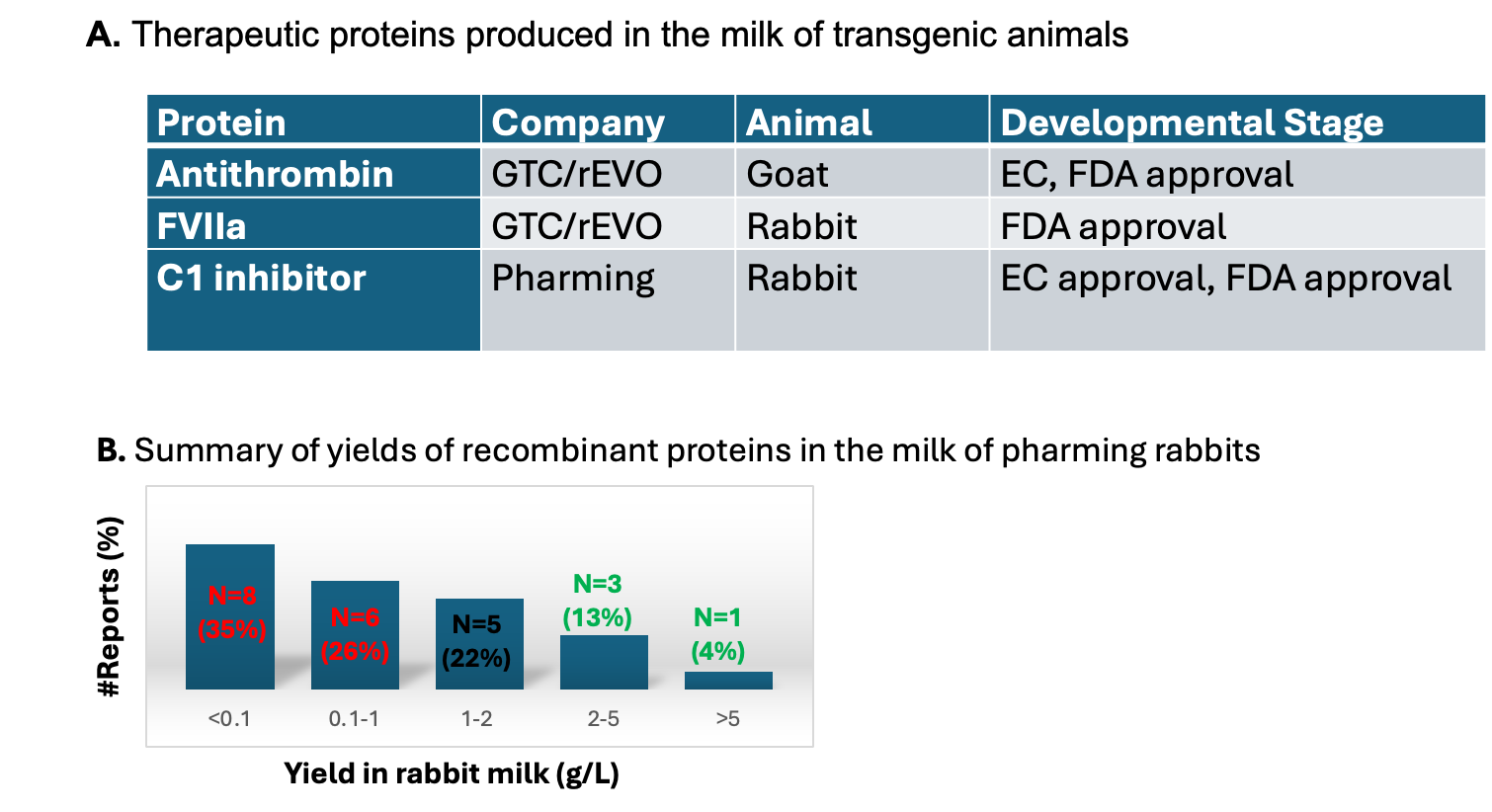
**

**Supplementary Figure 2. Illustration of the donor vector used to produce pharming rabbit founders.**

**
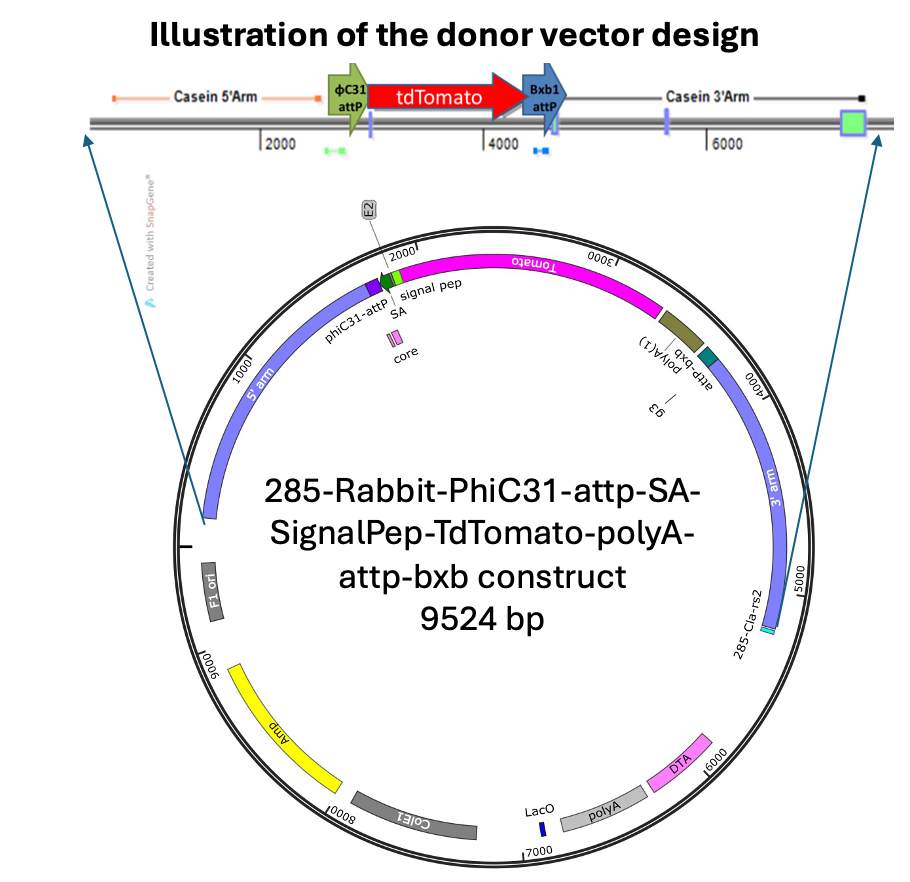
**

**Supplementary Figure 3. Sequence confirmation of a representative founder animal #84.** Both the 5’ PCR products and 3’ PCR products (see Figure 2C) were Sanger sequenced. The sequences are 100% matching the predicted sequences after successful knock-in at both the 5’ and the 3’ junctions.

**
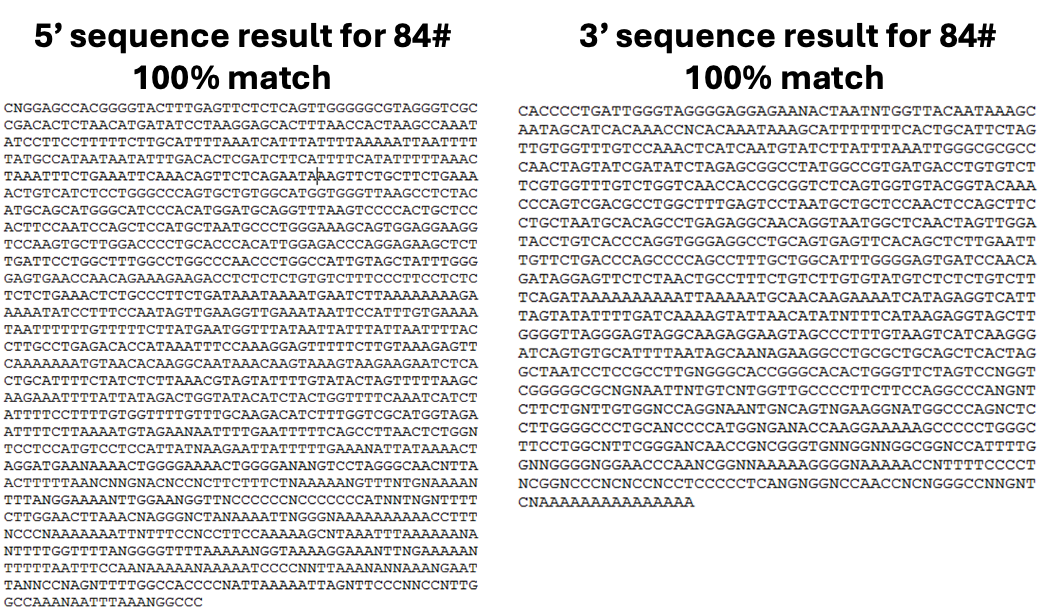
**

**Supplementary Figure 4. Determining the concentrations of recombinant tdTomato in the pharming rabbits.** (A) an example standard curve of the tdTomato Standards. (B) an example measurement of the concentration of tdTomato in the milk collected from a heterozygous female pharming rabbit.

**
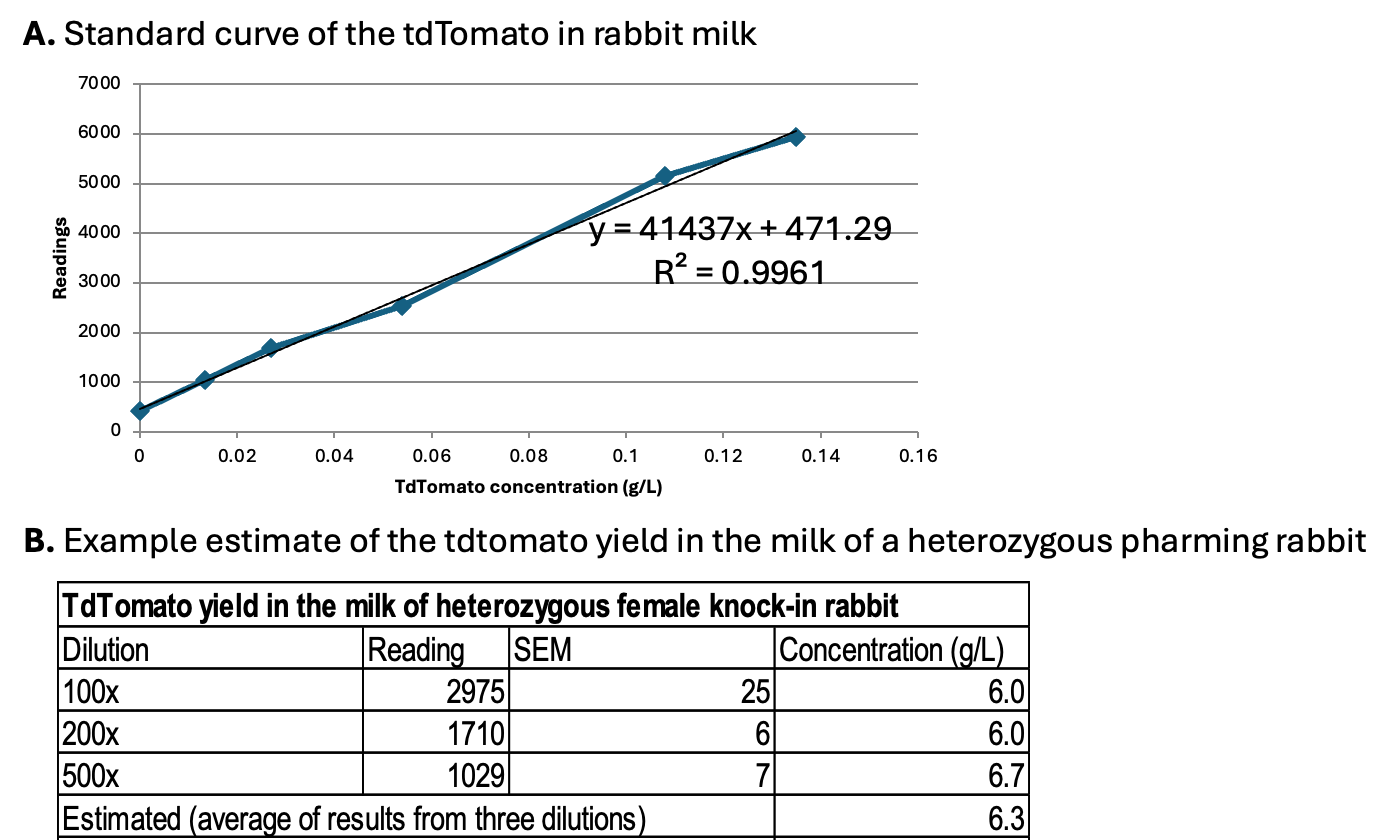
**

**Supplementary Figure 5. Illustration of the docking site ready (DSR) strategy to streamline the production of pharming rabbits.** The tdTomato expressing pharming rabbits, also referred to as DSR rabbits, produced in the present work carry integrase recognition sequences flanking the transgene tdTomato. In a future application, a donor template with the new gene of interest flanked by integrase sequences can be injected along with integrases to empower Cas9-free knock-in in a cassette exchange manner.


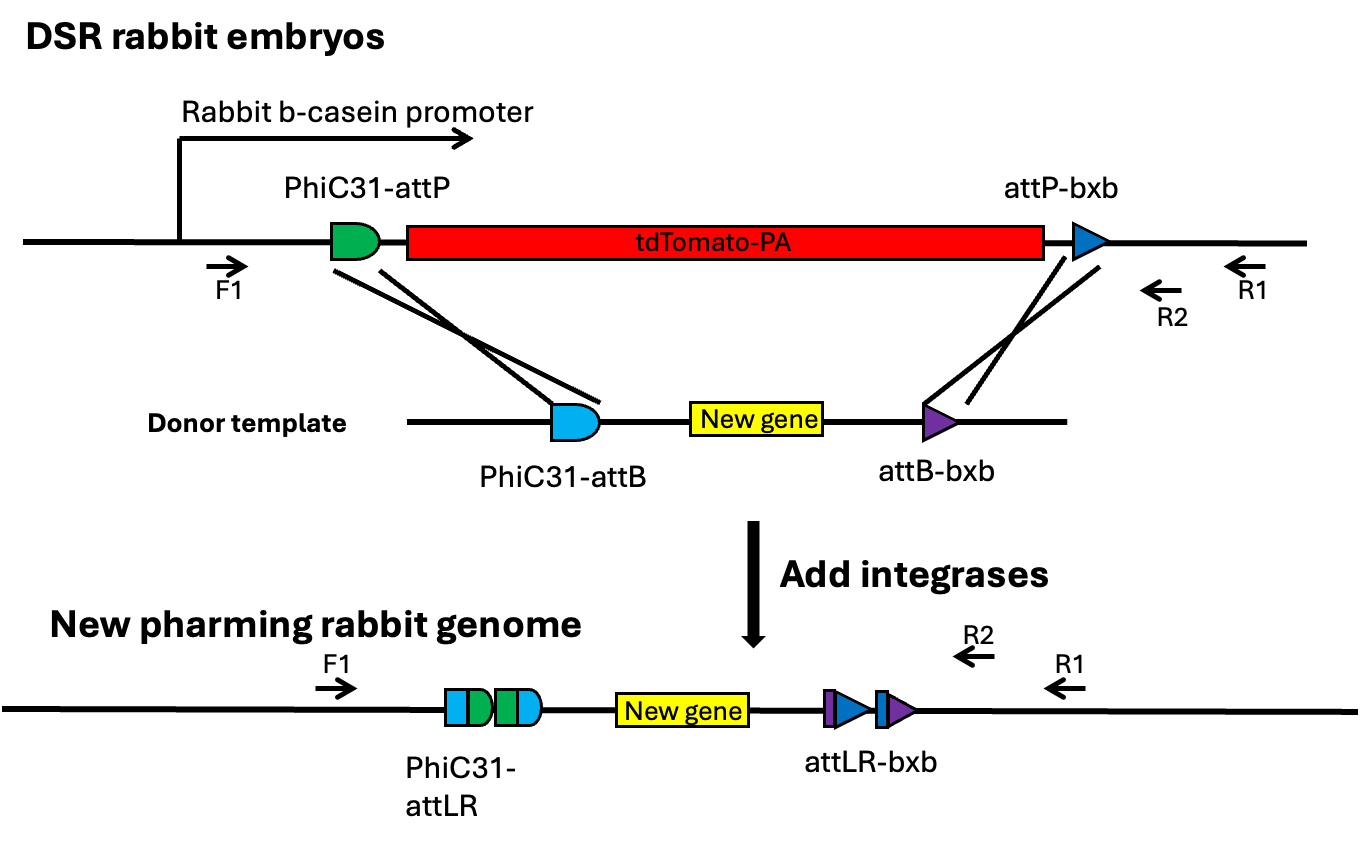

Supplement: Supplementary file 1 — Additional file1 (DOCX 1378 kb) [file 40659_2025_653_MOESM1_ESM.docx]
